# Applying next-generation sequencing to track *falciparum* malaria in sub-Saharan Africa

**DOI:** 10.1186/s12936-019-2880-1

**Published:** 2019-09-03

**Authors:** Sofonias K. Tessema, Jaishree Raman, Craig W. Duffy, Deus S. Ishengoma, Alfred Amambua-Ngwa, Bryan Greenhouse

**Affiliations:** 1 EPPIcenter Program, Division of HIV, Infectious Diseases, and Global Medicine, Department of Medicine, University of California San Francisco, San Francisco, CA USA; 2 Centre for Emerging Zoonotic and Parasitic Diseases, National Institute for Communicable Disease, Sandringham, Gauteng South Africa; 3 Department of Infection Biology, University of Liverpool, Liverpool, UK; 4 National Institute for Medical Research, Tanga Research Centre, Tanga, Tanzania; 5 MRC Gambia Unit, The Gambia at LSHTM, Fajara, The Gambia; 6 Chan Zuckerberg Biohub, San Francisco, CA USA

**Keywords:** Next-generation sequencing, Malaria genomics, Molecular epidemiology, Tracking parasites

## Abstract

Next-generation sequencing (NGS) technologies are increasingly being used to address a diverse range of biological and epidemiological questions. The current understanding of malaria transmission dynamics and parasite movement mainly relies on the analyses of epidemiologic data, e.g. case counts and self-reported travel history data. However, travel history data are often not routinely collected or are incomplete, lacking the necessary level of accuracy. Although genetic data from routinely collected field samples provides an unprecedented opportunity to track the spread of malaria parasites, it remains an underutilized resource for surveillance due to lack of local awareness and capacity, limited access to sensitive laboratory methods and associated computational tools and difficulty in interpreting genetic epidemiology data. In this review, the potential roles of NGS in better understanding of transmission patterns, accurately tracking parasite movement and addressing the emerging challenges of imported malaria in low transmission settings of sub-Saharan Africa are discussed. Furthermore, this review highlights the insights gained from malaria genomic research and challenges associated with integrating malaria genomics into existing surveillance tools to inform control and elimination strategies.

## Background

In 2017, there were approximately 198 million cases of malaria and 400,000 malaria-related deaths in sub-Saharan Africa (SSA) [1]. This burden is highly heterogeneous; 10 countries contributed 70% of the total reported cases [1], while others made substantial progress towards realizing elimination as an attainable goal [2]. This heterogeneous nature of malaria distribution in SSA has resulted in countries with very low transmission sharing borders with higher transmission countries, a scenario clearly evidenced in southern Africa [3]. On a finer scale, this phenomenon extends to neighboring regions within the same country, and has resulted in some SSA countries implementing subnational targeted malaria elimination strategies [4, 5].

**Fig. 1:**
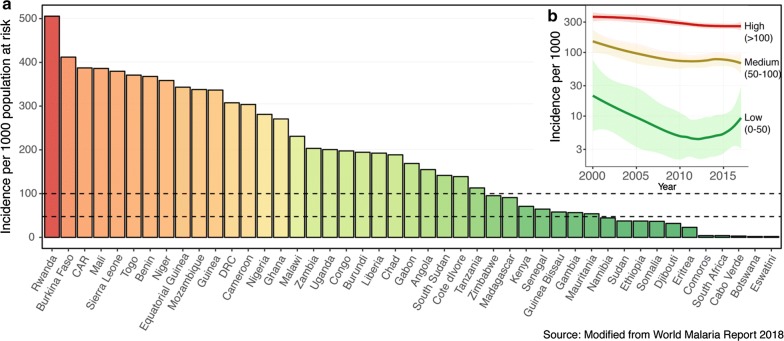
Variation in malaria transmission intensity in 45 sub-Saharan Africa countries (data from) (**a**). Distribution of malaria incidence in 2017. **b** Temporal change in the incidence of malaria in low (incidence 0–50 per 1000 population at risk), medium (incidence 50–100) and high (incidence > 100) transmission countries of SSA. Data source: http://apps.who.int/gho/data/node.imr.SDGMALARIA?lang=en

Spread of malaria from higher to lower transmission areas is not a new challenge, but has become a more pressing issue given the recent progress in malaria control and increasing connectivity between SSA countries driven by increased population movements [6]. In areas of low malaria transmission, an apparent “outbreak” of cases could merely arise from imported cases and could present a barrier to achieving malaria elimination [7] (Fig. 1). Imported malaria remains difficult to identify and address using the surveillance tools and limited resources currently available to most National Malaria Control Programmes (NMCPs). While existing surveillance measures (e.g. parasite rate, case incidence and reported travel histories) are fundamental to answering some of these questions, they are often limited by consistency and accuracy, particularly in areas with highly mobile and migrant populations.

Recent advances in malaria genomics, when combined with epidemiologic data, offer the potential to improve our ability to track the spread of malaria [8–12]. Several studies have highlighted the value of malaria genomics in epidemiological and public health settings (reviewed by [11–13]). Most of these studies have relied on traditional genotyping methods to understand transmission dynamics [14, 15]; to determine connectivity between populations [16–18]; to classify local and imported infections [19] and to track the spread of drug resistance as discussed by Ishengoma et al. in this technical series [20]. The increasing accessibility of high throughput next-generation sequencing (NGS) now provides an opportunity to refine existing tools and develop high resolution approaches [17, 21–27]. Despite this potential, molecular epidemiology approaches are not yet fully operationalized to inform intervention strategies in SSA. In this review, how malaria genomics can improve the understanding of malaria transmission dynamics by enabling the tracking of parasite flow between populations and its promise to address the challenges of identifying imported infections are discussed. This review highlights the major challenges in study design, gaps in laboratory and analytical methods and translating genomic data into calibrated epidemiologic parameters. Furthermore, issues surrounding logistics, local capacity, communication between researchers and NMCPs and the need to establish a framework for actionable genomic epidemiology studies in SSA are highlighted.

## Genomic epidemiology of malaria: from transmission to translation

### Estimating population level transmission from genomic data

Parasite genetic data from polymorphic loci (e.g. microsatellites and highly diverse genes); single nucleotide polymorphisms (SNP) and whole genome sequences (WGS) have been used to characterize transmission dynamics [11, 13, 14]. It is generally believed that as malaria transmission declines, complexity of infection and genetic diversity decreases, leading to spatial fragmentation of parasite populations [11, 28–31]. This association can ideally be leveraged to assess the level of ongoing local transmission and evaluate the impact of control or elimination interventions [32]. In this context, genomic data would augment traditional surveillance methods, and may be particularly useful in settings where surveillance infrastructure is limited or where traditional metrics are insensitive to relevant changes in transmission. Within-host and population level genetic indices have both been used to measure transmission intensity.

#### *Measuring within*-*host genetic indices*

Within-host parasite diversity can arise from sequential bites from mosquitoes that are infected with genetically distinct strains (superinfection) or more commonly from a single mosquito bite containing multiple strains, some of which may be closely related (co-transmission) [33]. Traditionally, within-host parasite diversity is measured by characterizing a few polymorphic loci to determine the minimum number of genetically distinct parasite strains present (i.e. multiplicity of infections, MOI) (reviewed by Zhong et al. [33]), but these methods have limited inter-laboratory reproducibility and comparability among studies. SNP panels and analytical tools have been developed to mitigate the limitations of size-polymorphic markers [15, 34–37]. However, SNPs begin to lose resolution in highly complex infections where most loci with reasonable diversity will result in heterozygous calls. Advances in NGS technologies have made it possible to develop highly sensitive targeted deep sequencing approaches [22, 38–43]. These data can provide information not only the number of strains, but also the genetic diversity [e.g. the within-host diversity index (*Fws*)] as well as relatedness and genetic structure of parasites in an infection [22, 43–46]. Multi-locus deep sequencing data can provide information on the number and genetic relatedness of strains in an infection, which can be utilized to evaluate spatial differences and temporal changes in transmission. A recent exciting development is the ability to sequence single infected red blood cells, providing high-resolution data, including the ability to understand drivers of genetic diversity within naturally occurring infections and to unambiguously phase genetic data from mixed infections [33, 47]. However, these methods are not yet easily scalable to the levels required for most epidemiologic work, requiring additional development.

In malaria-endemic regions, multiple infections are common, and within-host diversity indices broadly correlate with endemicity in a non-linear fashion [14, 28–32, 44, 48–50]. Reduced within-host diversity has been associated with increased ITN use [51] and temporal changes in transmission [50, 52, 53], indicating that theses metrics may be reasonable indicators of changes in transmission intensity. However, these relationships may be more difficult to interpret in areas where infections are not at a steady-state, and in particular may not reflect local transmission intensity, even amongst locally acquired infections, in areas with high rates of importation [54]. Although NGS technologies are advancing quantification of within-host diversity, they have also introduced new challenges. For example, the depth of sequencing can dramatically affect within-host diversity indices and comparability of findings between studies. Therefore, study design and the sequencing workflow including sample preparation, choice of sequencing platform, sequencing depth and sequence processing pipelines would benefit from standardization across molecular epidemiology studies. Similarly, the sensitivity of computational methods to identify genetic variants and associated errors are crucial for the successful establishment of NGS-based inference of transmission and the possibility to integrate these tools for routine surveillance.

#### Measuring population level genetic indices

While population level genetic diversity is complex, there are a range of genetic measures, including heterozygosity (or homozygosity), effective population size, linkage disequilibrium, proportions of genetically related infections and others [11, 12]. These indices have been traditionally generated using microsatellites and SNP panels. However, NGS offer the potential to provide data at a greater breadth to evaluate the genetic relatedness of parasites within and between infections. Several methods (such as DEploidIBD [44], DEploid [55], hmmIBD [56] and isoRelate [57]) are now available to identify genomic regions that are shared between isolates thereby detecting whether pairs of infections within and between populations are genetically related or not.

Several studies have compared the spatial differences in these indices and evaluated changes over time. Declining transmission has been associated with increase in linkage disequilibrium, reduction in parasite genetic diversity and effective population size likely as a result of population bottlenecks and fragmentation of parasite populations [11, 12]. However, the relationships between transmission intensity and changes in genomic indices are unlikely to be linear and uniform across settings. A recent study in the Kingdom of Eswatini [54], a country with a yearly malaria incidence of < 1 malaria case per 1000 population at risk, revealed a high level of parasite diversity. The authors found that this seemingly paradoxical finding was consistent with transmission driven by a high proportion of imported infections with a few, short chains of local transmission, a finding supported by the NMCP’s classification of most cases as imported based on travel history data. Findings such as this highlight the challenge of applying broad genetic relationships to scenarios where transmission is dynamic—with dramatic changes in transmission occurring over short spatial and/or temporal scales. Such areas may represent the rule rather than the exception in low and very low transmission settings of SSA where effective interventions may increase heterogeneity in transmission [16] Therefore, additional work is needed to determine sampling frames, relevant meta-data and appropriate statistical frameworks to obtain calibrated transmission metrics from genomic data such that these data can complement existing tools and improve the scale and resolution of population level inference of transmission.

### Inferring connectivity from parasite genetic data

Genetic data are often used to estimate connectivity between populations and track the spread of infectious diseases. In malaria, the genetic relatedness of infections can be compared at different scales to determine spatial patterns of transmission [8, 16, 22, 58, 59]. Infections with the same or highly similar genomes linked by epidemiologic data, are likely to represent infections that are related by transmission. The genetic resolution required to distinguish the relatedness of infections over a given number of transmission events depends on the underlying transmission intensity; the spatial scale of the study; sampling frameworks and parasite density (e.g. symptomatic and/or asymptomatic samples) and the ability of the assay to detect genetically distinct strains in polyclonal and low-density infections.

Over the years, several analytical methods to infer population structure have been developed [60–62]. However, genetic relatedness-based approaches have recently been shown to provide a greater resolution to identify geographic regions that are linked by transmission [16, 58, 59]. These approaches have the potential to define spatial patterns in low to moderate transmission areas. However, genetic relatedness metrics alone are not enough to accurately estimate importation; analytical tools to translate genetic connectivity to demographic connectivity (e.g. the number of parasites imported from one location to the other) are required. This is a major problem in most areas of SSA where human connectivity and parasite flow occurs between genetically intermixed populations. However, the availability of reference WGS data and resultant ability to inform geographically informative target selection frameworks targets, would improve the resolution of genetic data to accurately assign parasites to their geographic origin.

Beyond population level estimates of connectivity, parasite genomics offers enormous potential to determine person-to-person transmission chains [63]. These approaches are routine for viral and bacterial diseases [64, 65] but are not yet readily applicable for sexually recombining pathogens such as malaria. Tracking of infections using this approach is based on the genetic similarity between the ensemble of parasites present in each of a pair of infections representing a potential transmission event. Therefore, the accuracy of transmission chains is dependent on the genetic resolution (i.e. the informativeness of genetic targets and the sensitivity of genotyping methods); the proportion of infections sampled; the available epidemiologic meta-data; the analytical methods and the context within which these methods are applied. In SSA, where imported infections are often highly complex and diverse [16, 66], genotyping methods must be able to detect minority strains from low density infections by targeting regions of the genome that exhibit genetic diversity sufficient to provide individual strain identification in mixed infections.

### Classification of local and imported infections using parasite genetic data

Malaria importation poses a complex challenge in low transmission countries of SSA [7]. Despite the success of malaria control over the past decade, recent reports show an increase in the number of clinical cases in pre-elimination countries, which is mainly attributed to importation from neighboring higher transmission areas [1, 3] (Fig. 1b). These challenges are more pronounced in southern Africa, where malaria eliminating countries are being challenged by cross-border importation from higher transmission countries [3, 67, 68]. For example, more than 50% of malaria cases in Eswatini and South Africa were classified as imported based on reported travel history of patients [3, 67, 68]. Progress towards elimination requires novel approaches with improved accuracy to classify imported malaria and measure its impact on local transmission. Molecular epidemiology provides an as yet untapped opportunity to address these challenges.

NGS can provide high-throughput genomic data from routinely collected field samples (e.g. dried blood spots and used RDTs), however even the best genetic data alone cannot be expected to accurately reconstruct transmission chains. Availability of a toolbox of appropriate analytical methods that combine parasite genomics and epidemiological data [8], would be very useful for reconstruction of transmission chains and derivation of other transmission metrics. Furthermore, this approach can provide information on the number of secondary cases arising from each imported infection, allowing programmes to directly measure receptivity and determine what if any interventions are required to prevent re-establishment of transmission in an eliminating area. Therefore, a multidisciplinary team of modellers, molecular epidemiologists, programme officers and other stakeholders need to work together to fully harness the potential of malaria genomics to address the current and future challenges of imported malaria in very low transmission settings of SSA.

## Challenges and priorities for the translation of malaria genomic epidemiology

**Fig. 2:**
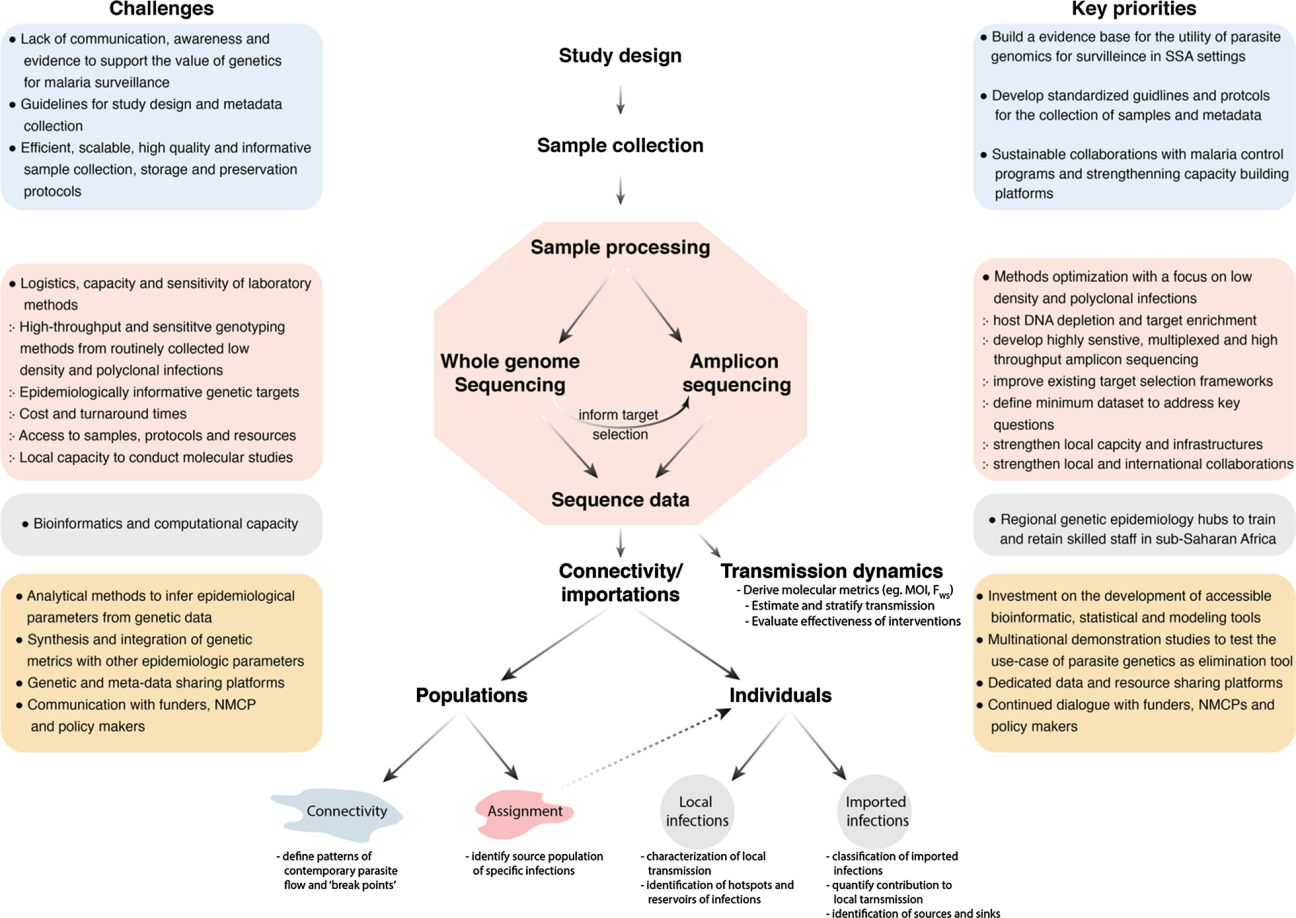
Challenges and key priorities of genomic epidemiology in sub-Saharan Africa

Genomic epidemiology has grown rapidly over the past decade, facilitated by decreasing costs, increased efficiency of NGS technologies as well as improved infrastructure for data management and analysis. These improved technologies have enabled researchers to explore how malaria genomics can help improve the understanding of transmission dynamics and flow of parasites. However, translation of findings to inform control and elimination and its applications in SSA has lagged behind other regions owing to a number of factors, some of which are illustrated in Fig. 2 and discussed below.

### Challenges of integrating with routine surveillance systems

Integration of genomic data into malaria surveillance is a relatively recent phenomenon, largely facilitated by the rapid increase in accessibility to these technologies. While this makes the field very exciting scientifically, there is limited evidence on the utility of these methods to provide timely and actionable data. There are even fewer data available to guide standardization or best practices on the types of sampling frames to consider, genomic data to generate, and analytical plans to employ in order to answer epidemiologically relevant questions. Although these types of evidence are rapidly being generated, it is understandably difficult for research groups and other stakeholders interested in evaluating or deploying these technologies to obtain buy in from the relevant field partners. Some NMCPs may not consider genomic epidemiology as an integral part of their suite of surveillance tools or even one worth investigating through operational research. For under-resourced NMCPs where achieving adequate coverage of basic control interventions is a major challenge, genomics may be viewed as a costly luxury that they can ill afford. What is often not appreciated by programmes and donors, and in truth is currently difficult to formally evaluate, is the cost of deploying (or not deploying) expensive interventions based on substandard surveillance data. Therefore, there is very urgent need for discussions and operationally relevant studies that highlight the value of genomic epidemiology. In addition, it is essential that standardized frameworks are created which enable the integration of genomic epidemiology into existing surveillance systems in low and very low transmission areas of SSA. The potential applications of genomic epidemiology depend on the timely, efficient collection of samples, as well as access to sample information and associated meta-data, which should be leveraged through existing surveillance programs such as health facility-based surveillance, malaria indicator surveys (MIS), therapeutic efficacy studies (TES) and others. These approaches provide cost-effective and scalable platforms, which help establish efficient and equitable collaboration with NMCPs while addressing programmatic questions without requiring much if any costs for additional sampling.

### Challenges with laboratory methods and data analyses

Two major challenges associated with laboratory-based analyses in SSA are the sensitivity of the technology required to generate high quality data from low-density infections and the detection of relevant strains in highly polyclonal infections. The ability to obtain epidemiologically useful information from the genomes of mixed infections is an issue in most of SSA, where polyclonal infections are often the rule rather than the exception even in relatively low transmission areas [16, 66, 69]. Advances in NGS have enabled the whole genome and targeted sequencing of malaria from routinely collected field samples [70], but no single method is universally applicable for all contexts. WGS can provide the near complete genome of the parasite, while targeted NGS can generate data at specific loci of interest. Although both approaches rely on a more or less similar laboratory workflow, the required quantity and quality of DNA; cost, laboratory processing, data storage, and computational time differ significantly. Unlike WGS, targeted sequencing provides greater depth of sequencing allowing for improved detection of minority strains in an infection and more robust comparisons between samples, since amplification of targeted loci is deeper and more consistent. However, identification of informative genomic regions and the capability to effectively multiplex these targets are challenges that need to be overcome to fully realize the utility of this method. Which and how many targets provide sufficient information to track the flow of parasites or determine their origin from different regions of SSA is currently unknown. Furthermore, it is appealing to develop combined panels to efficiently answer multiple questions, such as transmission epidemiology and prevalence of molecular markers of drug resistance. Therefore, optimization of sensitive laboratory methods and the development of well calibrated and accessible data analyses tools need to be prioritized and addressed.

### Challenges with local infrastructure and capacity

Despite the potential of NGS technologies, there are many challenging and costly bridges to cross in order to realize its full potential and application in SSA. The lack of local capacity in many SSA countries is the result of many complex issues, including limited infrastructure, the shortage of African expertise in malaria genomics and the lack of adequate support by African governments and international donors. There are efforts to overcome some of these limitations (e.g. The Malaria Genomic Epidemiology Network (MalariaGEN), The Plasmodium Diversity Network Africa (PDNA), MRCG-LSHTM genomics and high-performance computer centers and others), however a sustainable approach will, in part, depend on African scientists advocating for governments and international funders to build local facilities and establish sustainable collaborative platforms to train and attract skilled staff necessary to lead malaria genomics in SSA. One model would be to advocate for funding of regional genomics centers, which can provide services to geopolitical blocs. These would help to galvanize adoption and ensure sustainability, since local scientists and control program staff would be directly invested in the process and as such be able to better direct the outcome.

### Challenges with communication of findings

Genomic surveillance, coupled with high quality metadata, can provide valuable input for the development of policies for malaria elimination and eventual eradication. Critical to the success of any control/elimination programme is the involvement of and ownership by the respective local governments. For example, WHO-recommended TES have allowed strong partnership between NMCPs and researchers to plan, implement and report findings [71]. These partnerships also extend to the dissemination of findings to policy makers and development of policy recommendations, particularly when changes of first-line anti-malarials are required. Experience gained from such initiatives need to be formalized and utilized to develop efficient and sustainable partnerships between researchers and NMCPs for a broader range of molecular epidemiology studies. In the context of wide adoption and utilization of genomic data to support malaria elimination strategies, NMCPs should be capacitated on the added value and how genomic data can be integrated to inform routine activities of the programmes. The programmes also need to be supported and sensitized to appreciate how genetic data can potentially support and influence the process of changing different malaria control/elimination policies. Whenever possible, the NMCPs should play a central role in the planning and implementation of genomic studies in order to ensure that the research address questions of relevance and priority in the local context. Ensuring adoption of malaria control or elimination policies guided by research findings must be a joint effort of NMCP, researchers and other key stakeholders. Finally, the parasite genetic surveillance findings must be disseminated to key stakeholders and policy makers in language that they clearly understand. Making the data interpretable and accessible, e.g. by using maps and interactive visualization tools, must be a priority for genomics surveillance.

## Data sharing and accessibility

The cost of sequencing has dropped significantly, resulting in the rapid generation of genomic data by researchers from developing and developed countries. An important benefit of NGS data is that it can be shared and may be directly compared, even if the sequences were generated by different laboratories. Data sharing is essential for enabling and promoting malaria genomic research in a way that will maximize the outcomes from molecular epidemiology studies and provide resources for the development and validation of laboratory and analytical tools. There are encouraging efforts in data sharing by the malaria community (e.g. the pioneering efforts demonstrated by MalariaGEN and PlasmoDB). However, consistent deposition of sequence data from various scientists into standardized repositories is not yet coordinated at the scale seen in other fields. It is challenging to formulate efficient solutions for data standardization, formatting, archiving and access in the absence of a dedicated repository for malaria genomic data. In the context of SSA, limited access to high-speed internet and computational capacity presents a barrier for African researchers to access and utilize publicly available genomic data. In the future, platforms such as PlasmoDB or any newly designed dedicated data sharing platform must adequately address the challenges and bottlenecks in malaria genomic data sharing (e.g. ethical, administrative, logistic and data management issues) and their accessibility by SSA researchers.

## Conclusions and future perspectives

NGS technologies hold enormous potential for scientists and malaria control program personnel in SSA to improve their understanding of malaria transmission dynamics, including the ability to tracking the spread of parasites between populations or from one individual to the other. Ultimately, with sufficient reference genomes from multiple sites, it may be possible to accurately assign infections to their geographic origin. This information would help to address the formidable challenges of accurately evaluating imported malaria in low and very low transmission settings. It is clear that sensitive laboratory methods, appropriate analytical and dissemination tools, and accessible data sharing platforms need to be prioritized and developed to materialize the promises of malaria genomics, especially in SSA. Strategies for sample collection that leverage existing surveillance systems and, when appropriate, standardization of study designs will help to facilitate sustainable partnerships between researchers, NMCPs, and other stakeholders. African researchers need to play a leading role in advocating for international donors and African governments to build local capacity, possibly through regional genomic epidemiology hubs that can spearhead the generation of data to inform malaria elimination and eradication policies on the continent.
